# Assessing the Health Loss from Kashin-Beck Disease and Its Relationship with Environmental Selenium in Qamdo District of Tibet, China

**DOI:** 10.3390/ijerph18010011

**Published:** 2020-12-22

**Authors:** Jing Wang, Shengcheng Zhao, Linsheng Yang, Hongqiang Gong, Hairong Li, Cangjue Nima

**Affiliations:** 1 Key Laboratory for Geographical Process Analysis & Simulation, Research Institute of Sustainable Development, Central China Normal University, Wuhan 430079, China; jingwang@ccnu.edu.cn; 2 Key Laboratory of Land Surface Pattern and Simulation, Institute of Geographical Sciences and Natural Resources Research, Chinese Academy of Sciences, Beijing 100101, China; yangls@igsnrr.ac.cn; 3 Tibet Center of Disease Control and Prevention, Lhasa 850030, China; zhaoshengcheng_520@163.com (S.Z.); rockyghq@126.com (H.G.); nc916@126.com (C.N.); 4 College of Resources and Environment, University of Chinese Academy of Sciences, Beijing 100049, China

**Keywords:** Kashin-Beck disease, Tibet, health loss, years lived with disability, selenium

## Abstract

Kashin-Beck Disease (KBD) is one of major endemic diseases in China. In this study, we estimated the health loss from KBD in Qamdo district of Tibet using the years lived with disability (YLD) metric and investigated the influence of environmental selenium (Se) on it by multiple regression model. The results showed that YLD rates produced a different ranking of health loss of KBD from that produced by prevalence rates between Basu and Luolong County, with higher health loss from KBD (43.61 YLD/1000) but lower prevalence (17.86%) in Basu County. YLD rates in two counites were both highest for the 45–64 years age group. Compared with the prevalence rate, the YLD rate had a closer relation to environmental Se and was significantly negatively correlated with Se in both soil and highland barley. The multiple linear regression further revealed that Se contents in cultivated soil and highland barley were main influencing factors for the health loss of KBD, which could explain 90.5% of the variation in YLD rates. The information obtained highlights the significance of the YLD metric in exploring the environmental etiology of KBD and provides important information on which to base decisions on future prevention and control of endemic diseases.

## 1. Introduction

Kashin-Beck disease (KBD) is a chronic, multisite, deformative osteoarthropathy, which is characterized by necrosis and remodeling of cartilage including growth plates [1,2]. It mainly harms the growth and development of children and adolescents, involving multiple symptoms, such as the short fingers and limbs, the retarded growth, and disabilities in the advanced stages [3,4]. According to the standard of diagnosis of KBD (WS/T 207-2010), patients with multiple, symmetrical thickening of finger joints or deformity of short fingers/toes are diagnosed as clinical cases; patients whose hands present multiple symmetrical X-ray signs at bone end or metaphysis are diagnosed as X-ray positive cases. KBD is distributed diagonally from northeastern China to Tibet in the southwest, with additional endemic regions in neighboring areas of Russia and some regions of Vietnam and Korea [1,4]. Currently, it is still relatively active in Tibet since new clinical cases were detected occasionally in some affected counties [5]. According to the 2018 health statistics issued by the Chinese Ministry of Health [6], there were 535,878 individuals affected by KBD in 379 counties of 13 provinces or autonomous regions. This disease has been one of the major endemic diseases that severely impact the physical health and life quality of local residents and hinder the economic and social development of disease areas.

The severity of KBD is usually evaluated by prevalence rates or X-ray positive rates of children. These traditional indexes are often limited and unilateral and can only count the number of cases. By contrast, the years lived with disability (YLD), proposed by the World Bank and World Health Organization, takes the prevalence and severity of the disease into account simultaneously. YLD expresses the consequences of living with less than perfect health conditions. It is an estimate based on the length of time that a condition persisted along with any accompanying disability and thus is an indicator of the non-fatal burden of disease [7]. For a given disease, it can be derived by multiplying its prevalence rate by a disability weight, thus comprehensively reflecting the harm caused by the disease [8]. YLD was developed for the Global Burden of Disease Study (GBD) to measure the non-fatal disabling effect of diseases and injuries and has previously been used for the World Bank to inform priority setting for health research and optimize the allocation of health resources [8,9,10,11]. To the best of our knowledge, however, there is no research on the estimation of YLD for KBD. Little is known about the health loss caused by KBD.

The relationship between KBD and selenium (Se) has always been a hot topic in the environmental etiology of KBD. Numerous studies have demonstrated that the prevalence of KBD is closely related to the low Se environment [12,13,14,15,16,17], and Se supplementation can effectively control and prevent KBD [18,19,20,21]. Nevertheless, there were still some studies questioning this Se deficiency etiological hypothesis. They deemed that Se deficiency was not the direct and only cause of KBD since KBD did not occur in some areas where Se level was low, such as New Zealand [22] and Finland [23]. Generally, when exploring relationships of KBD with environmental Se, it is customary to use the prevalence rate of KBD. This metric is likely to provide incomplete information on the effect of different degrees of KBD. Since YLD can better value the harm caused by KBD, its correlation with environmental Se may be more conductive to the exploration of environmental etiology of KBD. The influence of environmental Se variables on the health loss from KBD also needs to be clarified.

Therefore, the health loss of KBD in Qamdo district of Tibet and its relationship with environmental Se were evaluated in this study. The main objectives were to 1) quantify the health loss from KBD among different groups of population in Qamdo district by adopting the YLD metric; 2) investigate how environmental Se variables are related to the health loss of KBD using the multiple regression model. We hypothesized that the correlation of environmental Se with YLD rate would be stronger than that with prevalence rate of KBD. Thus, the results may provide important evidence for the exploration of environmental etiology of KBD. The estimation of health loss from KBD will also be helpful for optimizing the allocation of health resources and identifying high risk population for future targeted prevention and intervention.

## 2. Materials and Methods

### 2.1. Study Area

**Figure 1:**
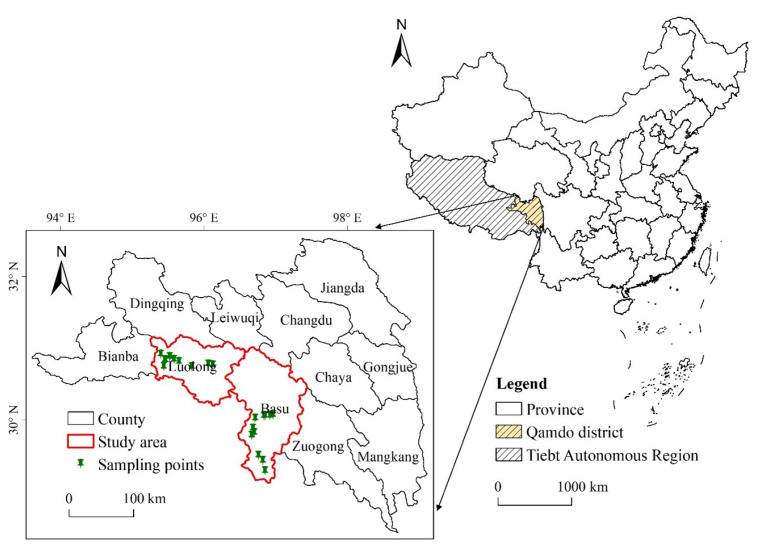
Location map for the study area and distribution of sampling sites.

Qamdo district, located in eastern Tibet within the Hengduan Mountain region and Three-River Valley, is one of the main KBD endemic areas in Tibet [24]. In 2014, Tibet Institute of Endemic Disease Prevention and Control, together with Qamdo Center for Disease Prevention and Control, monitored the prevalence of KBD in Qamdo district. The monitoring included clinical examinations among adults, and right-hand X-ray and clinical examination among children aged 7–16. Basu County and Luolong County as the most serious KBD endemic areas in Qamdo district were taken as the case study in this research (Figure 1). With the method of stratified cluster sampling, one township was first selected from the east, south, west, north, and middle of each county, and then one village was selected in these five directions of each township. Some investigation points were adjusted slightly due to the distribution of population and traffic problems in the rainy season [25]. Finally, a total of 19 monitoring sites were selected and investigated in the study area. Highland barley, tsamba, rice, and flour constitute the major food for the local residents. In particular, highland barley as the staple crop in Qamdo district contributes 78.7% of crop planting areas in 2014 [26]. Tsamba is also made of highland barley, butter, and tea. Rice and flour are usually exogenous staple food purchased from the market.

### 2.2. Study Populations

All residents in the selected monitoring sites were examined, with a total of 6119 people; 3072 of them were males and 3047 were females, which accounted for 50.2% and 49.8% of the total subjects. The age of the subjects ranged from 1 to 98 years old. There were 3946 adults aged 17 years old and over and 2173 children aged under 17 years old. The ratios of male to female were 0.95:1 for adults and 1.12:1 for children. The average age of adults and children were 40 years old and 9 years old. Among these populations, 5339 people underwent KBD clinical examination and 1434 of them were diagnosed as grade I or above. The prevalence rate of KBD in adults were 21.89% and 28.68% in Basu and Luolong County, respectively. In addition, 780 children aged 7–16 underwent X-ray examination and 38 of them were diagnosed by X-ray positive. The X-ray positive rate in children were 5.01% and 4.75% in Basu and Luolong County, respectively. The data were collected through person-to-person interviews and clinical examination. The detailed individual information including name, gender, age, and address were also recorded, and we used available de-identified data only. All subjects gave their informed consent for inclusion before they participated in the survey. The informed consent of children was obtained from their teachers and parents. The study was conducted in accordance with the Declaration of Helsinki, and the protocol was approved by the Ethics Committee of Tibet Institute of Endemic Disease Prevention and Control (No. 20140521).

### 2.3. Sampling and Analyses

Along with the disease surveillance group, a total of 321 environmental samples were collected from 19 KBD-affected villages in Basu and Luolong County in 2014. These samples included 26 natural soil (0–20 cm) (NS), 57 cultivated soil (0–20 cm) (CS), 40 highland barley (HB), 40 tsamba (TB), 40 rice, 33 flour, and 85 drinking water (DW). The location of sampling sites is shown in Figure 1. NS samples were taken from the land far away from villages, farmland, and roads, without or with less human disturbance. CS samples were taken from relatively large farmland away from villages or roads after removing the withered crop leftovers. Five sub-samples were taken from each farmland according to the diagonal method, which were then mixed together to create a composite sample. Staple food and DW samples were randomly collected from households. The determination of total Se referred to national standards on the determination of Se in foods (GB/T 5009.93-2003) and soil (NY/T 1104-2006). Additionally, 0.1 mol/L (pH = 7.0) KH_2_PO_4_-K_2_HPO_4_ buffer solution was used to extract Se in soil available fractions (SAF). Details of the method can be found in Xu et al. [27]. Se contents in all samples were determined by hydrogen generation-atomic fluorescence spectrometry (AFS-9780, HaiGuang Instruments, Beijing, China), whose detection limit is 0.02 ng/ml and RSD is less than 1.0%. Reagent blanks, duplicated samples, and national standard reference materials (GBW10011 for wheat and GBW07410 for Tibetan soil) were used for analytical quality control.

### 2.4. Calculation of YLDs and YLD Rate

The YLDs can be calculated from either an incidence perspective or a prevalence perspective. The former is the product of incidence, disability weights and average duration of disease; the latter is the product of prevalence of disease and disability weights, which is convenient for comparison with the recent GBD studies [28]. In this study, we calculated the prevalence-based YLDs for the analysis. Given the long duration, non-fatal outcomes, and late-onset serious symptoms of KBD, which is quite similar to the endemic diseases such as schistosomiasis and goiter, we intended to adopt the same strategy carried out in the GBD study of these two diseases. Namely, the duration of the disease was hypothesized as one year [29,30], and thus there is no discounting problem. To be consistent with GBD study, we did not apply age weighting in estimating YLDs.

The epidemiological data of KBD was collected from the aforementioned disease surveillance in 2014, which was provided by the Tibet Institute of Endemic Disease Prevention and Control of Tibet Center of Disease Control and Prevention. In the light of the examination results, the specific prevalence rates of KBD by age group and gender were calculated. The population data for Basu and Luolong counties in 2014 was calculated based on the 2010 population census of Tibet Autonomous Region. According to the Tibet statistical yearbook in 2015, the total population growth rate of Qamdo district between 2010 and 2014 was 3.97% [31]. Age was divided into five groups: 5-, 10-, 15-, 45-, 65 years and over for hierarchical summary and comparison.

Disability weights (DW), representing the magnitude of health loss associated with specific health outcomes, are important parameters for calculating YLDs. They are usually estimated based on evaluation scales [28,32] or referenced from the results of Global Burden of Disease (GBD) studies [33,34]. No paper has previously reported the disability weight of KBD. Nevertheless, the clinical manifestations of KBD are quite similar to those of rheumatoid arthritis, which mainly include pain, limited motion, and deformities of joints [35,36]. Therefore, in this study, we directly adopted the disability weights of rheumatoid arthritis in the latest GBD 2017 study. The disability weights of KBD in different grades were assigned as 0.117 (grade I), 0.317 (grade II), 0.581 (grade III), respectively, in accordance with the sequela of rheumatoid arthritis [37].

Finally, on the basis of the above prevalence and demographic data, YLDs and YLD rates (YLD/1000) of KBD in Basu and Luolong counties are calculated by gender and age groups using the GBD YLD template for prevalence YLD [38].

### 2.5. Multiple Linear Regression Model

To investigate how Se in the environment affects the health loss from KBD, the multiple linear regression (MLR) analysis was performed for YLD rate with Se contents in various environmental media as multiple independent variables. In this work, environmental Se variables includes Se contents in CS, NS, SAF, HB, TB, rice, flour, and DW. Kolmogorov-Smirnov’s test was first used to detect the normality of variables. The relationship between YLD rate and environmental Se variables was then modeled in the way of stepwise multiple regression. The stepping criteria employed for entry and removal were based on the significance level of the F-value and set at 0.05. Stepwise MLR constructs a multivariate model for the dependent variable, *Y*, based on a few deliberately selected explanatory variables. The equation takes the following form:

(1) $$Y=a_{0}+a_{1}X_{1}+a_{2}X_{2}+\cdots +a_{n}X_{n}$$

where *Y* is the dependent variable (i.e., the YLD rate); *X*_1_, *X*_2_, …, *X*_n_ are the independent variables (i.e., Se contents in CS, NS, SAF, HB, TB, rice, flour, and DW); $a_{0}\;$is the constant, where the regression line intercepts the Y axis. $a_{i}$ (1≤i≤n) is the standard partial regression coefficient. Equation (1) is used to investigate which variables influence its response and at what extent. The results were checked for multi-collinearity by examining the variance inflation factors (VIF) of predictor variables. For a reliable MLR analysis, VIF for all the independent variables should be less than 10 [39,40]. SPSS 26.0 software (IBM Corp., Armonk, NY, USA) was used for statistical analysis of data and development of MLR model. The statistical significance was set at the 0.05 level.

## 3. Results

### 3.1. YLDs and YLD Rate of KBD

Table 1 showed the calculated YLDs and YLD rate in different KBD grades by gender. The total health loss from KBD in 2014 was estimated at 1769.30 YLDs (43.61 YLD/1000) in Basu County and 1656.98 YLDs (33.56 YLD/1000) in Luolong County. There was no significant difference between the two genders (*p* > 0.1). KBD of grade III (654.69 YLDs, 37.0% of total YLDs) contributed most to the total YLDs in Basu; while in Luolong, 77.4% of total YLDs were due to KBD of grade I (1282.69 YLDs). When YLDs were compared with prevalence rate as a measure of disease burden, there was no consistently corresponding relationship. The prevalence of KBD in Basu County (17.86%) was far lower than that in Luolong County (27.06%), whereas the burden of disability caused by KBD in Basu County (43.61 YLD/1000) was higher than that in Luolong County (33.56 YLD/1000).

### 3.2. Patterns by Age Groups

**Figure 2:**
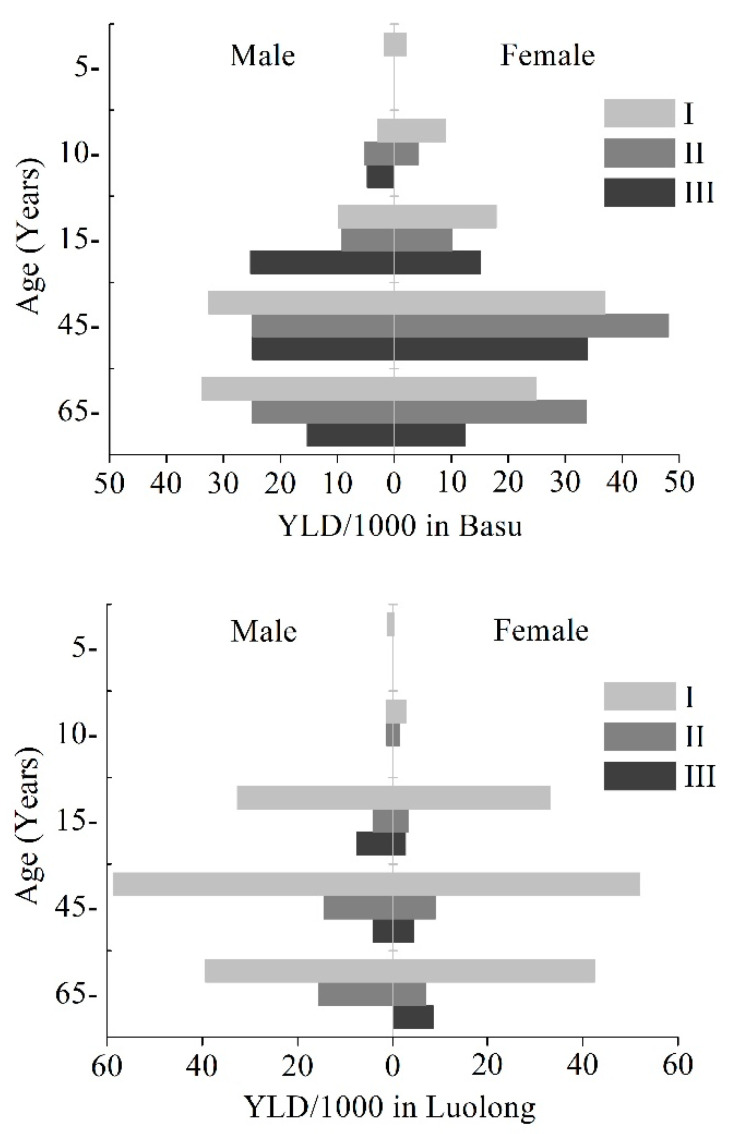
Age-specific YLD rate by gender and KBD grades in Basu and Luolong County.

Figure 2 displayed the comparison of age-specific health loss of KBD between genders and KBD grades in two counties. As a whole, YLD rates in two counties both increased with age and decreased at the highest 15–44 age group. YLD rates in Basu County were relatively higher for all age groups than those in Luolong County. The highest YLD rate in Basu was found in female aged 45–64 and with KBD of grade II (48.20 YLD/1000), while that in Luolong was found in male aged 45–64 and with KBD of grade I (58.64 YLD/1000). For each age group in Basu County, there was no significant difference in YLD rates among different grades of KBD. However, in Luolong County, YLD rates in KBD of grade I were significantly higher than other grades for all age groups (*p* < 0.05).

### 3.3. Se Content in the Environment

Table 2 summarized the total Se contents in various environmental samples and soil available Se in the study area. It showed considerable variations in total Se of topsoil, ranging from 61.8 to 462.6 μg/kg, with higher average Se content in CS (250.9 ± 83.8 (57) μg/kg). However, this total Se level is still lower than the background value of national A-layer soil (290 ± 255 μg/kg) of China [14]. The average total Se of NS in Luolong County was significantly higher than that in Basu County (*p* = 0.003), while there was no significant difference in CS and SAF between the two counties. Se contents of self-produced foodstuff (i.e., HB and TB) were obviously lower than those of purchased grain (i.e., rice and flour). Except for HB and rice, significant differences of Se were observed in TB (*p* = 0.007), flour (*p* = 0.008) and DW (*p* = 0.000) between two counties. Lower average Se contents of flour in Luolong might be due to the fact that some flour samples were from locally self-produced wheat. Se content of HB in Luolong was slightly lower than that in Basu County, although the former had higher soil Se content. This might be related to the lower available Se in soil of Luolong County.

### 3.4. Correlation Analysis

Kolmogorov-Smirnov’s test showed that all variables satisfied the normal distribution (Sig. > 0.05). Pearson correlation matrix of various variables in this study was shown in Table 3. Statistically significant correlation coefficients were highlighted in bold. YLD rates were negatively correlated with Se contents in all environmental samples. Significant correlations were observed with Se contents in NS (r = −0.628), CS (r = −0.853), and HB (r = −0.727). Similarly, the prevalence rate of KBD was also negatively correlated with environmental Se, but significantly correlated with Se in HB (r = −0.837), TB (r = −0.541) and DW (r = −0.534). The cross correlation between different environmental Se variables were also showed. For instance, positive and significant correlations were found between NS and CS (r = 0.552), HB and TB (r = 0.768), as well as DW and HB (r = 0.707), TB (r = 0.598), as expected.

### 3.5. MLR Modeling

In order to obtain the most consistently significant regression equation for estimating the health loss of KBD, the data were put through a stepwise multiple regression with environmental Se contents as potential independent variables. The validity of the MLR model was determined based on the F-test and its associated significance level. As shown in Table 4, the significance (Sig.) level of the model is 0.001 showing that the model offers a good fit for the data. The coefficient of multiple determination, R^2^, for the model is 0.905, suggesting that 90.5% of the variation in the YLD rate can be explained by independent variables listed in the table. The most important influencing factors for the YLD rate are Se contents in cultivated soil and highland barley. Other environmental Se variables were eliminated from the model due to their relatively weak correlations. The VIF values of the two variables were 1.315 < 10, indicating that there is no multicollinearity between the independent variables in the regression model.

## 4. Discussion

In the present study, we estimated the health loss from KBD in Qamdo district using the YLD metric and investigated the influence of environmental Se on the YLD rate of KBD. The epidemiological data of KBD used for calculating YLDs was based on field investigation, which is relatively complete and reliable. The calculation of prevalence and YLDs by gender and age groups in Basu and Luolong County was in accordance with methods in the GBD study [8], thus ensuring the authenticity, reliability, and comparability of the research results.

As far as the health loss from KBD is concerned, although the prevalence of KBD in Basu County is lower than that in Luolong County, the disability burden caused by KBD is higher in Basu County. This may be related to the distribution of the severity of KBD in two counties. It was found that 34.4% and 7.9% of patients were diagnosed as KBD of grade II and III in Basu and Luolong County, respectively, indicating that the health loss due to KBD of grade II and III in Basu are relatively high. One of the relevant contributions of using YLDs is that they give disability weights in different degrees according to the severity of KBD. Consequently, the ranking of the burden of disease differs from the ranking based on prevalence rate. Similar findings can be found in many studies on the burden of disease [41,42,43,44]. This result also suggests that the annual surveillance of KBD, only focusing on the prevalence or X-ray positive rate, may neglect the impact of serious disability caused by KBD on human health, which in turn affects the effective allocation of limited health resources. Attention should also be paid to the health loss caused by the disease.

When exploring the relationship of KBD with environmental Se, we compared the difference between the YLD rate and prevalence rate of KBD. The correlation analysis showed that the YLD rate of KBD was significantly and negatively correlated with Se in both topsoil and highland barley, while the prevalence rate was only significantly correlated with highland barley Se. Although the prevalence rate of KBD was also significantly correlated with Se in DW, an increase of Se in drinking water only plays a slight role in alleviating or preventing KBD due to its overall low concentration [16]. The average Se content of drinking water in the study area was only 0.63 ± 0.37 (85) μg/L (Table 2). Supposing that the water intake of an adult is 2 L per day, the amount of Se intake through drinking water is less than 3% of the recommended dietary allowance for healthy human adults (55 μg Se per day) [45], which is negligible. This result is in line with our previous assumption that the YLD rate of KBD has a closer correlation with environmental Se than its prevalence rate. It is probably attributed to the fact that YLD rate comprehensively reflects the harm caused by different degrees of KBD by simultaneously taking its prevalence and severity into account. Additionally, the YLD rate and prevalence rate were both significantly correlated with Se in self-produced HB, but not with Se in purchased rice and flour, indicating that residents in KBD endemic areas of Qamdo district were still highly dependent on their home-grown food crops. The results of stepwise MLR model further showed that, among all environmental Se variables, Se contents in cultivated soil and highland barley had the most important influence on the health loss from KBD in Qamdo district, which accounted for 90.5% of the total variation in YLD rates. This is expected since highland barley is the major food crops of local residents and soil is the ultimate source of Se in all foodstuffs. These results affirm that low Se content in natural environment is the major reason leading to the health loss from KBD.

Some limitations should be noted in the present study. First, reliable sources of disability weight are required to calculate YLDs. In this study, disability weights of different sequela of KBD were based on the results of rheumatoid arthritis in GBD 2017. It was assumed that these data would be acceptable as the two diseases have similar symptoms according to the health state lay descriptions in GBD 2017 disability weights dataset [37]. Another limitation of this study is the consideration of age weighting. The original GBD study weighted a healthy life lived at very young and old ages lower than other ages [46]. However, this is still controversial, since not all such studies agree that the youngest and oldest ages should be given less weight, nor do they agree on the relative magnitude of the differences [43,44,47]. Thus, the social values were not considered in this study. Despite these methodologic and data limitations, we believe that YLDs provide important information on which to base decisions on public health priorities. Third, some socio-economic factors, such as family income, educational attainment, etc., may also affect the incidence of KBD at the individual level, thus affecting the health loss of KBD. In the present study, we did not consider these factors, but mainly focused on the ecological relevance of the health loss from KBD with environmental Se. This is primarily because there was little difference in these socio-economic factors among villages we investigated. In future studies, stratified research can be considered by integrating individual factors with environmental factors at village level. The interactive effect of natural and social factors at different levels on the health loss of KBD will be further analyzed.

## 5. Conclusions

In China, although the prevalence of KBD has reached the level of elimination or control in the majority of affected areas, the loss of health life due to KBD should not be ignored. This is the first study that has attempted to use the method of GBD study to measure and compare the health loss from KBD among different population groups in Qamdo district of Tibet and investigate its relationship with Se in the environment. The findings suggest that YLD rates produce a different ranking of disease burden from that produced by prevalence rates alone. Considerable disparities in the health loss from KBD across age groups were observed in different regions of Qamdo. Compared with the traditional prevalence rate index, YLD rates have a closer relation to environmental Se. In particular, Se contents in cultivated soil and highland barley are main influencing factors for the health loss from KBD. The results obtained in this study can be used by policy makers for the targeting of prevention and treatment of KBD and optimizing the allocation of health care resources. The YLDs metric also represent a promising new tool for improving the ability of endemic disease prevention and control departments and other health agencies to assess and prioritize population health needs.

## Figures and Tables

**Table 1:** Health loss from KBD in Basu and Luolong County (2014).

County	KBD Grades	Male		Female		Total		Prevalence (%)
		YLDs	YLD/1000	YLDs	YLD/1000	YLDs	YLD/1000	
**Basu**	I	249.92	12.15	349.77	17.49	599.69	14.78	11.71
	II	212.49	10.33	302.42	15.13	514.91	12.69	3.83
	III	379.65	18.45	275.04	13.76	654.69	16.14	2.31
	Total	842.06	40.93	927.24	46.37	1769.30	43.61	17.86
**Luolong**	I	651.57	26.13	631.12	25.83	1282.69	25.98	24.92
	II	121.76	4.88	84.32	3.45	206.08	4.17	1.53
	III	108.14	4.34	60.07	2.46	168.21	3.41	0.62
	Total	881.48	35.34	775.51	31.74	1656.98	33.56	27.06

Abbreviations: KBD, Kashin-Beck disease; YLD, years lived with disability. KBD grades refer to the severity of KBD: grade I (mild), grade II (moderate), grade III (severe). The specific grading criteria are according to the diagnosis standard of KBD (WS/T 207-2010).

**Table 2:** Se contents of environmental samples in the study area (mean ± SD).

County	NS (μg/kg)	CS (μg/kg)	SAF (μg/kg)	HB (μg/kg)	TB (μg/kg)	Rice (μg/kg)	Flour (μg/kg)	DW (μg/L)
Basu	143.0 ± 54.4 (16)	245.3 ± 91.0 (36)	15.0 ± 4.3 (16)	9.0 ± 4.3 (26)	13.1 ± 7.4 (26)	49.8 ± 16.9 (26)	27.5 ± 10.8 (22)	0.79 ± 0.32 (39)
Luolong	207.6 ± 37.1 (10)	260.3 ± 71.0 (21)	13.7 ± 3.1 (14)	6.9 ± 2.9 (14)	6.4 ± 4.8 (14)	50.7 ± 16.3 (14)	16.9 ± 10.3 (11)	0.49 ± 0.35 (46)
Total	167.8 ± 57.4 (26)	250.9 ± 83.8 (57)	14.5 ± 3.7 (30)	8.4 ± 4.0 (40)	11.2 ± 7.1 (40)	50.0 ± 16.5 (40)	22.6 ± 9.1 (33)	0.63 ± 0.37 (85)

Abbreviations: NS = natural soil; CS = cultivated soil; SAF = soil available fraction; HB = highland barley; TB = tsamba; DW = drinking water. Numbers in parentheses are samples collected.

**Table 3:** Pearson correlation matrix of different variables.

	YLD Rate	Prev	NS	CS	SAF	HB	TB	Rice	Flour	DW
YLD rate (%)	1.000									
Prev (%)	0.718 **	1.000								
NS (µg/kg)	−0.628 *	−0.171	1.000							
CS (µg/kg)	−0.853 **	−0.518	0.552 *	1.000						
SAF (µg/kg)	−0.429	−0.241	0.390	0.447	1.000					
HB (µg/kg)	−0.727 **	−0.837 **	0.155	0.236	0.195	1.000				
TB (µg/kg)	−0.454	−0.541 *	−0.193	−0.008	−0.255	0.768 **	1.000			
Rice (µg/kg)	−0.168	−0.327	0.181	0.023	−0.159	0.431	0.332	1.000		
Flour (µg/kg)	−0.142	−0.433	−0.315	−0.273	−0.116	0.547	0.542	0.558	1.000	
DW (µg/L)	−0.371	−0.534 *	−0.114	0.125	−0.171	0.707 **	0.598 *	0.057	0.436	1.000

Abbreviations: YLD rate = years lived with disability per 1000 population; Prev = prevalence; NS = natural soil; CS = cultivated soil; SAF = soil available fraction; HB = highland barley; TB = tsamba; DW = drinking water. Numbers in bold are statistically significant coefficients. ** and * indicate that the correlations are significant at *p* < 0.01 and *p* < 0.05, respectively.

**Table 4:** Summary of multiple linear regression (MLR) model results for estimating YLD rates.

Independent Variables	Coefficients	Standard Error		Sig.		VIF
Intercept	97.848	8.882		0.000		
CS	−0.178 **	0.042		0.005		1.315
HB	−2.603 *	0.781		0.016		1.315
R^2^	0.905		Adjusted R^2^		0.873	
F	28.442		Sig.		0.001	

** *p* < 0.01; * *p* < 0.05. Note, VIF is variance inflation factor; Sig. is significance; CS is cultivated soil; HB is highland barley; R^2^ is the coefficient of multiple determination; F is the F value of F-test.

## Data Availability

The data presented in this study are available on request from the corresponding author and with permission of the Tibet Center of Disease Control and Prevention. The data are not publicly available due to confidentiality requirements.
